# Donor variability in HIV binding to peripheral blood mononuclear cells

**DOI:** 10.1186/1743-422X-5-95

**Published:** 2008-08-15

**Authors:** Joshua J Anzinger, Gene G Olinger, Gregory T Spear

**Affiliations:** 1 Section of Experimental Atherosclerosis, National Heart, Lung, and Blood Institute, National Institutes of Health, 10 Center Drive, Building 10, Room 5N111, Bethesda, Maryland, 20892, USA; 2 US Army Medical Research Institute of Infectious Diseases, 1425 Porter Street, Fort Detrick, Maryland, 21702, USA; 3 Department of Immunology and Microbiology, Rush University Medical Center, 1735 West Harrison Street, Cohn Building, Room 626, Chicago, Illinois, 60612, USA

## Abstract

**Background:**

HIV infection of cells varies greatly between individuals, with multiple steps in the replication cycle potentially contributing to the variability. Although entry and post-entry variability of HIV infection levels in cells has been demonstrated, variability in HIV binding has not been examined. In this study, we examined variability of HIV binding to peripheral blood mononuclear cells (PBMC) from different donors.

**Results:**

HIV binding to PBMC varied up to 3.9-fold between individuals and was independent of CD4. Replication of HIV in donor PBMC required CD4 and paralleled virus binding trends of donor PBMC. To assess the stability of virus binding phenotypes over time, HIV was bound to donors with low- and high-binding phenotypes. The binding phenotypes were maintained when tested weekly over a 4-week period for 3 of 4 donors, while one high-binding donor decreased to lower binding on the 4th week. The low- and high-binding phenotypes were also preserved across different HIV strains. Experiments performed to determine if there was an association between HIV binding levels and specific cell subset levels within PBMC showed no correlation, suggesting that HIV binds to multiple cell subsets.

**Conclusion:**

These results show that differences exist in HIV binding to donor PBMC. Our data also show that HIV binding to donor PBMC is CD4-independent and can change over time, suggesting that virus binding variability is due to differences in the expression of changeable cell-surface host factors. Taken together, this study highlights the impact of cell-surface factors in HIV binding to, and infection of, PBMC which likely represents an important step in HIV infection *in vivo*.

## Background

Infection of cells by HIV requires a number of host cell factors [1]. Differences in these factors can contribute to variability in infection levels between individuals. Previous studies show that HIV infection levels of cells differ between individuals, with up to a 1000-fold variability in replication for HIV laboratory-adapted strains [2,3] and up to a 40-fold variability for HIV primary isolates [4]. The level of HIV infectivity in cells has been attributed to multiple steps in the HIV replication cycle, including entry and several post-entry events [5]. These studies show that differences in host factors between individuals can impact the efficiency of HIV infection.

During the HIV replication cycle nascent virions acquire host cell-surface molecules that can retain their biological function. For example, CD54 (ICAM-1) incorporated in the virus membrane has been shown to facilitate attachment to cells independently of CD4 [6]. Additional studies have shown HIV attachment independent of CD4: macrophages can capture HIV through the mannose receptor and syndecans [7,8], dendritic cells through syndecan-3 and a variety of C-type lectin receptors [9-11], and HeLa cells through cell-surface heparans [12]. Furthermore, we previously showed that the initial attachment of HIV primary isolates to PBMC is independent of CD4 and not attributable to host factors previously suggested to be involved in HIV attachment to cells [13-15].

Since previous studies suggested that the initial attachment of HIV to PBMC is attributable to host factors, and that these factors could potentially differ between donor cells, we hypothesized that HIV attachment variability exists between donor cells. Heretofore, it has only been indirectly shown that variability in HIV entry exists between donor cells [5], however, no study has directly examined variability in HIV attachment to donor cells. In this study, we directly tested HIV attachment to donor PBMC and show high levels of variability in HIV binding to donor PBMC that occurs independently of CD4 and virus strain.

## Results

**Figure 1:**
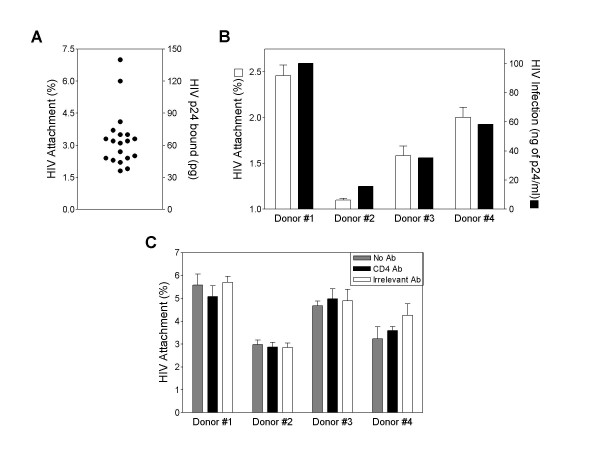
**HIV binding to PBMC is variable between donors**. **(A) **Freshly isolated PBMC (3 × 10^6^) were incubated with HIV_TH _(2000 pg of p24) in serum-free RPMI-1640 medium for 45 minutes on ice. After incubation with HIV, cells were washed to remove unbound virus. Cells were pelleted, lysed and HIV p24 was measured by ELISA. Shown are values from 19 different healthy PBMC donors. **(B) **HIV_TH _was bound to freshly isolated or PHA-stimulated donor PBMC. For virus binding to freshly isolated PBMC, HIV p24 was measured by ELISA as described above. HIV replication of donor PBMC was assessed by p24 ELISA after 5 days of culture. **(C) **HIV_TH _was bound to freshly isolated PBMC either in the presence of anti-CD4, isotype control antibody, or in the absence of antibody. Cell-bound virus was measured as described above. Results are the means ± SD of triplicate determinations.

We assessed the possibility of differences in HIV binding to donor PBMC using the R5 primary isolate HIV_TH _and freshly isolated PBMC from 19 different healthy donors. HIV binding was variable between donors, with up to a 3.9-fold difference in virus binding between the highest and lowest binding donors (Fig. 1A).

We next hypothesized that the variability in HIV binding would affect infection of PBMC. Donor PBMC from low, medium and high binding donors were treated with phytohemagglutinin (PHA) for 2 days, HIV was bound to PBMC and the cells cultured for up to 7 days. Replication of virus at day 5 post-infection showed a similar trend to virus bound to donor PBMC on day 0 (Fig. 1B). A similar trend was observed on day 7 post-infection (data not shown), although higher levels of replication were observed, as anticipated. As a control, virus was bound to PBMC in the presence or absence of PHA stimulation; there was no difference in virus binding between groups (data not shown), showing that PHA does not alter PBMC binding phenotypes.

While previous studies showed that binding of HIV primary isolates to PBMC was CD4-independent [10-12], we next sought to confirm this with PBMC donors that bound HIV at differing levels. The CD4-blocking antibody Sim.4 did not significantly affect binding of HIV to PBMC (Fig. 1C). regardless of whether the PBMC donor bound relatively high levels of virus (e.g. donor 1) or low levels of virus (e.g. donor 2). To confirm that the Sim.4 antibody could block infection, HIV was bound to PBMC at in the presence of Sim.4, an irrelevant antibody, or without antibody, and then incubated at 37°C to allow infection of PBMC to proceed. HIV infection of PBMC was inhibited with Sim.4, but not the irrelevant antibody (data not shown).

**Figure 2:**
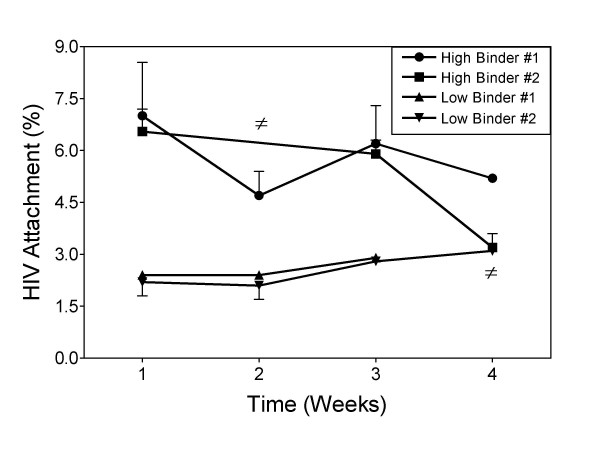
**HIV attachment phenotypes of PBMC donors are stable over time**. Freshly isolated PBMC from two donors with high-binding phenotypes and two with low-binding phenotypes were incubated with 2000 pg of p24 (HIV_TH_) in serum-free medium 45 minutes on ice and bound virus assessed as before. Donors that were not available are indicated by ≠.

We next determined whether HIV binding phenotypes of PBMC donors were stable over time. HIV attachment to PBMC from two donors with a high-binding phenotype and two with a low-binding phenotype were examined for virus binding levels once a week over 4 weeks. A stable HIV binding pattern was maintained over 4 weeks for PBMC from both of the donors with a low-binding phenotype and for one of the donors with a high-binding phenotype (Fig. 2). However, one donor with a high-binding phenotype changed to a low-binder on the fourth week. This data shows that HIV binding phenotypes are relatively stable over the period of several weeks, but are also able to change.

**Figure 3:**
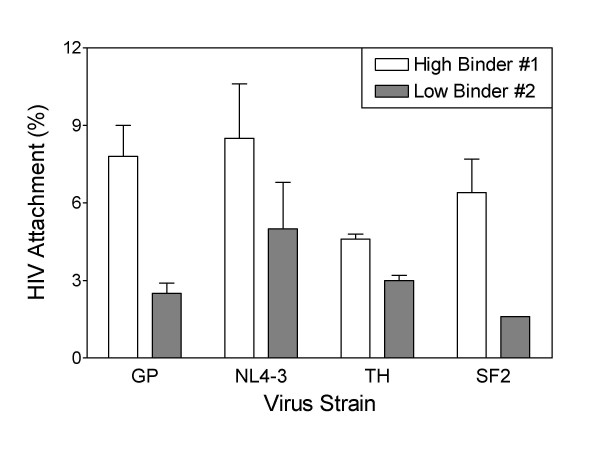
**HIV binding phenotypes of PBMC donors are maintained across multiple virus strains**. Freshly isolated PBMC were incubated with 2000 pg of p24 (HIV_GP_, HIV_NL4-3_, HIV_TH_, or HIV_SF2_) in serum-free medium for 45 minutes on ice. After incubation with HIV, cells were washed to remove unattached virus. Cells and bound virus were lysed and HIV p24 was measured by ELISA. Results are the means ± SD of triplicate determinations.

Since HIV infection of PBMC is affected by differences in virus strain [16], we also determined whether the relative binding of HIV to high- and low-binding PBMC was affected by the strain of virus. Four different HIV strains – HIV_GP _(X4), HIV_NL4-3 _(X4), HIV_TH _(R5), or HIV_SF2 _(X4) – were incubated with PBMC from one donor with a high-binding phenotype and one with a low-binding phenotype. The high-binding PBMC bound more virus than the low-binding PBMC for all virus strains tested (Fig. 3). Specifically, the high-binder PBMC bound 3.1-, 1.7-, 1.5- and 3.9-fold more virus than the low-binder PBMC for HIV_GP_, HIV_NL4-3_, HIV_TH_, and HIV_SF2_, respectively. This shows that HIV binding differences of high- and low-binding PBMC donors are preserved across multiple virus strains. Since the HIV binding variability between PBMC donors is not HIV strain-dependent, this suggests a role in cell binding of either host cell-acquired molecules on the HIV membrane, or a highly conserved HIV-encoded envelope protein site.

**Figure 4:**
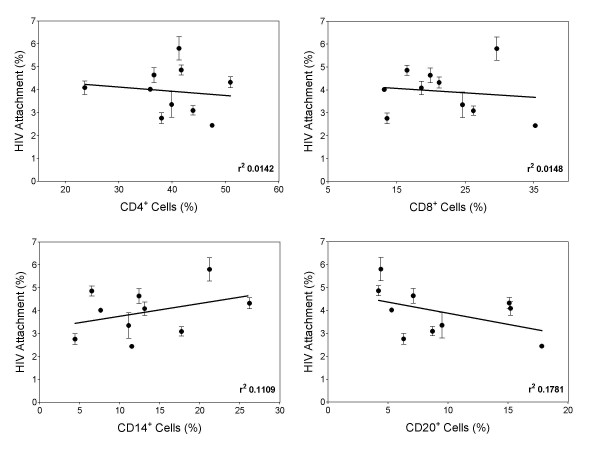
**Donor binding variability does not correlate with a specific cell subset within PBMC**. Donor PBMC were stained with anti-CD4-FITC, anti-CD8-FITC, anti-CD14-FITC, or anti-CD20-FITC and the percentage of each subset measured by flow cytometry. PBMC from donors were also incubated with 2000 pg of p24 (HIV_TH_), and cell-bound virus was measured by ELISA as before. HIV binding variability is plotted on the y-axis and percentage marker positive cell subsets is plotted on the x-axis. r^2 ^represents the goodness of fit value. Data represent means ± SD of triplicate determinations.

PBMC are a heterogeneous population of cells composed primarily of CD4 T cells, CD8 T cells, monocytes, and B cells. To determine whether donor binding variability is caused by higher or lower numbers of specific cell subsets within PBMC, HIV was bound to PBMC from 10 different donors, and flow cytometry was used to assess the percentage of CD4+ T cells (CD4+), CD8+ T cells (CD8+), monocytes (CD14+), and B cells (CD20+), respectively (Fig. 4). No correlation was observed between the level of virus binding and the percentage of CD4^+^, CD8^+^, CD14^+^, or CD20^+ ^cells, suggesting that virus binding variability between PBMC donors is due to differences in HIV attachment factors found on multiple types of cells, as opposed to differences in levels of a particular cell subset.

## Discussion

In this study we found that differences exist in the level of HIV binding to donor PBMC, that binding phenotypes of the donors are stable over four weeks, and that binding phenotypes are consistent across several HIV strains. Importantly, the variability in virus binding to cells correlated with the levels of HIV infection of cells. Ciuffi *et al*. reported that infection of cells from different donors was highly variable, and that 42% of the infection variance between cell donors was at the level of HIV entry [5]. The expression of CCR5 on CD4 T cells correlated with HIV infection levels of these cells [5], but much of this correlation was due to donors that were heterozygous or homozygous for the CCR5Δ32 allele. However, when donors that did not have the mutant allele were excluded from analysis, there remained a greater-than 10-fold difference in infection between individuals and about 50% of this was estimated to be due to virus binding and entry events, suggesting that cell-surface molecules can have large effects on virus infection of cells. While previous studies indicate the initial attachment of HIV to cells occurs independently of CD4 [10-12] and prior to gp120 interaction with CCR5 [17], we confirm in this study that the variability of HIV binding to donor PBMC is unaffected by the presence of a blocking antibody to CD4, indicating that the differences observed in the initial attachment of HIV to donor PBMC occurs by cell-surface molecules other than CD4.

Although the mechanism of HIV binding variability to donor PBMC has not been identified, studies performed in our laboratory show that the initial attachment of HIV to PBMC occurs in a Ca^2+^-dependent manner [13], suggesting a potential role for a C-type lectin or other Ca^2+^-dependent molecules in HIV binding to PBMC. Fusion of HIV mediated by HIV gp120/gp41 to permissive cells does not occur until 10 minutes after virus is added to cells at 37°C [18] and does not take place at 20°C, while binding of HIV can occur at 4°C or higher temperatures [14]. Therefore, the binding we observe is likely prior to fusion, and antibody inhibition experiments show it is independent of CD4. Donor binding variability has important implications for the HIV replication cycle, since infection of cells by HIV is greatly impacted by the amount of bound virus during either direct infection of cells or during transfer of virus between cells [15].

## Conclusion

By showing that high and low HIV-binding phenotypes exist, these studies highlight the impact of host-acquired molecules on the initial steps of HIV infection. This study therefore provides added insight into understanding the mechanism of HIV tethering to cells, which could potentially lead to the development of drug strategies that inhibit entry of the virus into cells.

## Methods

### Cell culture

PBMC were obtained from healthy donors by Ficoll-Hypaque (Whittaker M.A. Bioproducts, Walkersville, MD) gradient centrifugation of freshly obtained heparanized blood. To activate PBMC, 3 μg/ml phytohemagglutinin (PHA-L; Sigma/Aldrich, St. Louis, MO) was added to cells in RPMI 1640 medium supplemented with 10% heat inactivated fetal bovine serum and 50 μg/ml gentamicin (complete medium). PBMC were cultured for 2 days, washed, and further cultured in complete medium containing 20 U/ml interleukin-2 (obtained through the AIDS Research and Reference Reagent Program [ARRRP], National Institute of Allergy and Infectious Diseases, National Institutes of Health, from Maurice Gately, Hoffman-La Roche, Inc.). All virus strains tested were produced in PHA-stimulated PBMC of healthy donors as previously described [19].

### HIV binding

PBMC (3 × 10^6 ^PHA-stimulated or unstimulated) were incubated with 100 μl of virus-culture supernatant, diluted in serum-free RPMI to contain 2000 pg of p24 for 45 min on ice with periodic agitation. After incubation with HIV, 3 ml of ice-cold phosphate buffered saline (PBS) was added to cells. Cells were pelleted by centrifugation and then transferred to a new tube and washed to prevent measurement of HIV binding to tubes. Pelleted cells were lysed with 0.5% Triton X-100, and the amount of virus bound to cells was measured by p24 antigen ELISA (National Institutes of Health AIDS Vaccine Program, Frederick, MD). For experiments with blocking antibody, either anti-CD4 (Clone Sim.4, obtained from American Type Culture Collection, ATCC; IgG_1_), an isotype control antibody (Clone G18-145, ATCC; IgG_1_), or no antibody was added to tubes at the beginning of the virus/PBMC incubation step.

### HIV infection

For infection of PBMC, freshly isolated PBMC were cultured in complete medium with 3 μg/ml PHA for 48 h and then washed prior to HIV binding. Cells were incubated with HIV and washed as described above. PBMC were then transferred to 24-well plates (Corning Inc., Corning, NY) and cultured in medium with 20 U/ml IL-2 in a total volume of 1 ml. HIV p24 in culture supernatants was measured by ELISA on days 5 and 7. Cultures were fed on days 5 and 7 by removal of 500 μl and replacement with fresh medium containing IL-2.

### Flow cytometry

All antibodies (anti-CD4, -CD8, -CD14, -CD20 and isotype control) were FITC-conjugated mouse anti-human IgG (Becton Dickinson, Franklin Lakes, New Jersey). To assess the expression of cell-surface markers, PBMC were incubated with the manufacturer's recommended volume of antibody on ice for 30 min, and then assessed for surface-markers by flow cytometry. For each PBMC donor, the percentage of surface-marker positive cells was plotted against the amount of HIV bound to donor PBMC.

## Competing interests

The authors declare that they have no competing interests.

## Authors' contributions

JJA and GGO performed all of the experiments. JJA participated in writing of the manuscript. GTS provided overall direction and co-wrote the manuscript. All authors read and approved the final manuscript.
